# Reports on Hospitals of the United Kingdom

**Published:** 1914-08-01

**Authors:** Henry Burdett


REPORTS ON

### Hospitals of the United Kingdom.

By SIR HENRY BURDETT, K.C.B., K.C.V.O.

SERIES III.

### THREE LEICESTER HOSPITALS.

### (1) THE FAIRE HOSPITAL.

This hospital has been open for two years and is intended to meet the needs of the working men and working women who are disinclined to accept the charity of the voluntary hospitals but who cannot afford the charge of a nursing home. The wish of members of the working classes in an increasing degree in Leicester is to obtain the best surgical skill and the most efficient nursing on payment—*i.e.*, for a comparatively small sum within their means. If the actual condition, equipment, efficiency, and comforts of the Faire Hospital could be brought home to the knowledge and minds of the majority of the working classes in Leicester, there can be little doubt that it would prove one of those institutions which would very seldom have a vacant bed. The applications for admission are steadily increasing, and we hope that it may be found possible in the near future to take such steps as will secure that the facilities offered by this hospital are made known directly to the head of every working man's family throughout the world.

The hospital contains three wards, which are attractive in appearance, well proportioned, and equipped in so excellent a manner as to exhibit marked intelligence, knowledge, and generosity on the part of Mr. Faire and his advisers. For two years the hospital has been run, through the generosity of Mr. John E. Faire, on the basis of charging the patients 25s. per week and an additional 5s. for the use of the theatre. Medical attendance is paid for by the patients in accordance with the arrangement made between themselves and the practitioner in attendance, most patients selecting their own family doctor to take charge of them or the members of their family when they are patients at this institution. The rule is that no fee for an operation shall exceed ten guineas, and in practice it appears that the surgical and medical fees are extremely moderate having regard to the quality of the service and the attractions, comfort, and efficiency of the hospital in all departments so far as the patients are concerned.

The whole hospital has been equipped regardless of expense. We found the hospital in excellent condition. The interest taken in it, not only by the committee and members of the staff, under the inspiring direction of the founder, but also by the matron and her assistants, was evidenced everywhere, and must make this little hospital an ideal place, we should say, for members of the working class who have the independence and intelligence to become patients as and when occasion arises.

We gathered that the actual receipts from patients are not enough at present to defray the whole cost of the hospital's maintenance. This is not surprising having regard to the fact that 25s. a week is not a large enough sum to meet all the outgoing expenses entailed by the maintenance of a bed in a modern up-to-date hospital such as the Faire Hospital can properly claim to be. Still it is such an asset for the working classes that we believe it has only to become more widely known to make it financially successful, providing the thrift basis on which it is founded is extended by changing the rules of admission so as to provide that all patients shall pay 25s. a week together with the 10s. or 7s. 6d. per week which they may receive as insured persons. The hospital is too good, and the idea which led to its foundation too sound, to suffer it to be in practice in any sense dependent upon charity for its upkeep. Leicester is to be congratulated upon the possession of the Faire Hospital, which represents a new type of institution, conceived in the best interests of the working classes, and sure, we are confident, increasingly to command their support and appreciation everywhere throughout the county.

### (2) THE MATERNITY HOSPITAL.

The Leicester Maternity Hospital is not only a hospital, but a district maternity institution. The latter portion of the work is carried on under the direction of a district midwife. The number of patients in 1913 was 342; each patient contributed 30s., and we should say from the appearance of the patients during the time of our visit that they are drawn from a fairly prosperous class of the working community. Midwifery pupils are taken, and eight of them successfully passed the examination of the Central Midwives Board in 1913. Five of them were sent by the County Nursing Association. There is evidence that the supply of pupils is diminishing considerably—a fact which renders the working of the institution difficult and expensive. Maternity nurses are also trained, six of them having obtained the hospital certificate of efficiency. The work of the hospital is at present carried on in a number of small cottages, and each patient has the advantage of occupying a separate room. Structurally it is in no sense a hospital, but as far as the patients are concerned the accommodation provided and the privacy obtainable must prove attractive features. This, we think, is evidenced by the fact that so small an institution should have admitted 342 in-patients for treatment during last year. Efforts are being made to raise a fund to purchase the freehold, and should they prove successful we presume that in due course plans will be invited, and a modern up-to-date hospital will eventually be built. The expenses of running this institution appear to be fairly large, a fact which we should take to point to the desirability of pushing forward the building fund energetically, in order to secure the erection of a new hospital on modern lines.

### (3) THE HIGHFIELD HOSPITAL.

This hospital is another instance of thrift development in the town of Leicester in the provision of medical treatment and skilled nursing. It is administered on a principle which is being increasingly recognised—namely, a graduated system of charges to suit the means of the patient. It is, in fact, a nursing home, with skilled attendance, for many who formerly had to depend for treatment upon a large institution at public cost. A few private nursing cases are admitted at a weekly fee of from two to five guineas, according to the accommodation, so that its benefits may be open to residents in Leicester who have somewhat ampler resources than the ordinary wage-earner. Humbler residents pay an amount which their resources permit them, though not the whole cost of their treatment in this hospital. The principle of admission is that every patient who presents an in-patient's letter or subscriber's recommendation is charged 6s. a week. All other patients, who apply without a subscriber's recommendation, pay 30s. per week; but this 30s., as in the case of the Faire Hospital, is not found to be sufficient to cover entirely the necessary expenditure entailed by their treatment and maintenance as in-patients. The last balance-sheet shows, we are glad to see, that the patients' payments exceeded one-half the cost of maintenance in this hospital during 1913. We cannot see why the hospital should not take the insurance money which insured persons receive while they are inmates of the hospital. Were this modification of the rules to be adopted it would appear from the accounts to be highly probable that the present annual deficiency would disappear, and that very shortly afterwards an encouraging surplus would be shown year by year. At the present time the hospital consists of a villa residence, which is in process of being modified and reconstructed, so that as funds permit an efficient hospital may be evolved in its entirety. The wards and equipment are attractive and good, but the nature of the building has not made it possible to supply adequate lavatory and other accommodation which it is hoped to secure in the near future, when the present temporary operation-theatre is reconstructed and brought up to date. In this hospital we were pleased to see evidence of an excellent spirit, and think it highly probable that the patients have good ground to be contented with their lot when they are fortunate enough to find themselves under treatment within its walls. We should be glad to see this institution developed on thrift lines until it becomes entirely independent of charity in any form or shape.

